# Novel transcription-induced fusion RNAs in prostate cancer

**DOI:** 10.18632/oncotarget.17099

**Published:** 2017-04-13

**Authors:** Sen Zhao, Marthe Løvf, Kristina Totland Carm, Anne Cathrine Bakken, Andreas M. Hoff, Rolf I. Skotheim

**Affiliations:** ^1^ Department of Molecular Oncology, Institute for Cancer Research, Oslo University Hospital-Norwegian Radium Hospital, Oslo, Norway; ^2^ Center for Cancer Biomedicine, Faculty of Medicine, University of Oslo, Oslo, Norway; ^3^ Department of Informatics, Faculty of Natural Science and Mathematics, University of Oslo, Oslo Norway

**Keywords:** prostate cancer, fusion transcript, RNA sequencing, expression profile

## Abstract

Prostate cancer is a clinically and pathologically heterogeneous disease with a broad spectrum of molecular abnormalities in the genome and transcriptome. One key feature is the involvement of chromosomal rearrangements creating fusion genes. Recent RNA-sequencing technology has uncovered that fusions which are not caused by chromosomal rearrangements, but rather meditated at transcription level, are common in both healthy and diseased cells. Such fusion transcripts have been proven highly associated with prostate cancer development and progression. To discover novel fusion transcripts, we analyzed RNA sequencing data from 44 primary prostate tumors and matched benign tissues from The Cancer Genome Atlas. Twenty-one high-confident candidates were significantly enriched in malignant *vs*. benign samples. Thirteen of the candidates have not previously been described in prostate cancer, and among them, five long intergenic non-coding RNAs are involved as fusion partners. Their expressions were validated in 50 additional prostate tumor samples and seven prostate cancer cell lines. For four fusion transcripts, we found a positive correlation between their expression and the expression of the 3′ partner gene. Among these, differential exon usage and qRT-PCR analyses in particular support that *SLC45A3-ELK4* is mediated by an RNA polymerase read-through mechanism.

## INTRODUCTION

Prostate cancer is world-wide the second most frequently diagnosed cancer type and the sixth leading cause of cancer-related death in men [1]. In the past decade, several discoveries of genetic alterations and gene expression abnormalities have revealed important molecular understanding to prostate cancer development and progression [2–5]. One predominant finding is the common expression of fusion genes in prostate cancer [5–7]. The most well-known is the fusion between *TMPRSS2* and *ERG*, which are present in nearly half of the prostate cancers [5]. Also several other ETS transcription factors are recurrently overexpressed, where these are juxtaposed to strongly expressed genes [8–10]. The upstream fusion partners may or may not be regulated by androgens [9, 11]. A number of common fusion transcripts generated from chromosomal rearrangements (*e.g*. translocation, insertion, deletion and inversion) have been detected by application of various high-throughput technologies [3, 4, 12–15].

Fusion transcripts, also known as chimeric RNAs, can also be generated without chromosomal rearrangements. For example, RNA polymerase read-through between two neighboring genes encoded on the same DNA strand and/or *trans*-splicing of pre-mRNA (*i.e*. two separated pre-mRNAs are spliced together and form a fusion transcript) are two mechanisms that give rise to fusion transcripts [16–18]. Unlike the classical fusion gene *TMPRSS2-ERG*, these fusion transcripts are the result of RNA processing and recombination without evidence of rearrangements on the DNA-level. Recent transcriptome-based analyses have revealed that transcription-induced gene fusions are commonly present in normal cells and tissues [14, 19, 20, 46]. Many of them probably represent stochastic events with little or no impact on cellular functions, whereas others, such as *SLC45A3-ELK4* and *TMEM79-SGM5*, have showed a high association with prostate cancer and indicate involvement in pathological processes [21, 22]. The use of next-generation sequencing technologies has advanced the identification of novel fusion transcripts in cancer samples, but it is still challenging to distinguish oncogenic driver fusions from passenger aberrations [13, 22–24]. The fact that fusion transcripts are present both in malignant and benign tissues provides an additional level of molecular complexity to consider in the search for biomolecules which are relevant to prostate cancer development, detection and treatment. Previous studies have only to a limited degree sought to capture the aberrant expression profile of fusion transcripts in cancer tissues [17, 22, 25, 26].

In this study, we aimed to identify recurrent and overexpressed fusion transcripts in prostate cancer. To achieve this, we analyzed the whole transcriptome sequencing data of 44 pairs of primary prostate tumors and adjacent normal tissues generated by The Cancer Genome Atlas (TCGA) [3]. Following the nomination of recurrent and overexpressed fusion transcripts, their expression patterns were further evaluated and validated using RNA sequencing data from 50 additional prostate tumor samples and seven prostate cancer cell lines. The inter-patient tumor-heterogeneity was explored by hierarchical clustering of the expression profiles of the fusion transcripts across the cohorts. Overall, our results revealed a complex landscape of novel and overexpressed fusion transcripts in prostate cancer. These transcripts probably represent a new repertoire for the discovery of novel cancer biomarkers and therapeutic targets.

## RESULTS

### Fusion transcript discovery

**Figure 1:**
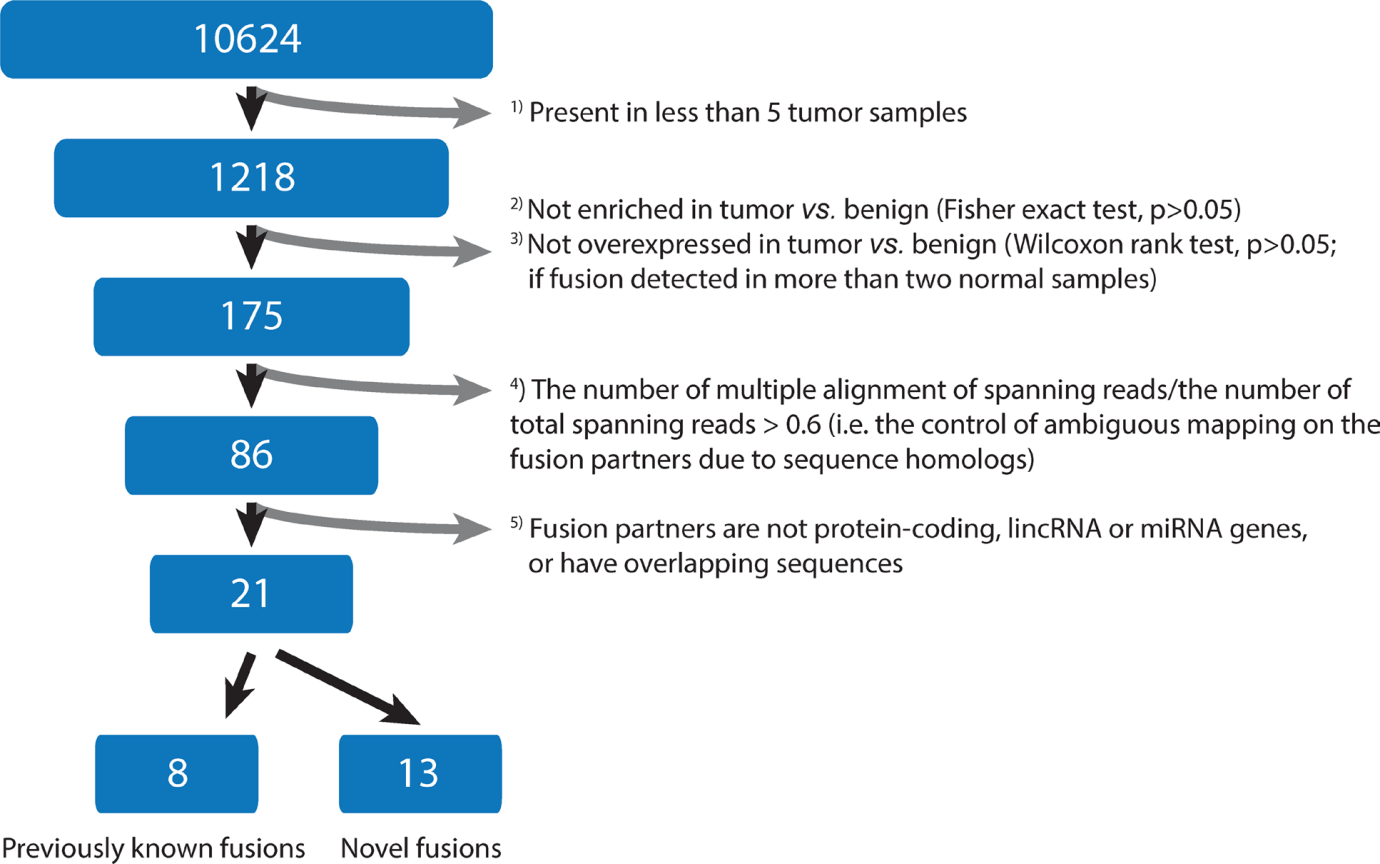
Flowchart of fusion transcript filtering The identified fusion transcripts were filtered in a successive manner, following the initial analysis of 44 paired tumor and benign prostate samples by deFuse software.

To identify fusion transcripts, we analyzed RNA-seq data from TCGA prostate cancers by use of deFuse software. Initially, deFuse predicted 10,624 fusion transcripts across 44 paired tumor and benign prostate samples, and 1,218 candidates were considered further as they showed positive signals in at least five tumor samples (Figure 1). Of these, 175 fusion transcripts with both a significant enrichment of detected number (*p* < 0.05, Fisher's exact test; see Materials and Methods) and overexpression level in tumor *vs*. benign (*p* < 0.05, Wilcoxon rank-test) were retained for further analysis. To control the false-positive identification of fusion partners due to sequence homology, 89 fusion transcripts with a large proportion (> 0.6) of multiple spanning reads to total spanning reads were removed. This reduced the candidates to 86. Furthermore, 60 fusion transcripts whose partner genes are related to overlapping and pseudo-genes were filtered out (Figure 1). Altogether, a set of 21 reliable fusion transcripts was finally nominated (Table 1). Analysis of the Illumina body map RNA-seq dataset (ArrayExpress accession ID E-MTAB-513 and European Nucleotide Archive accession ID ERP000546) shows that none of the 21 fusion transcripts are detected from RNA-seq data of 12 human healthy tissues, which also included one sample of prostatic tissue. However, in the TCGA data, expressions of the 21 fusion transcripts were detected at various levels in 0 to 30 of the 44 benign tissue samples (Table 1). Hierarchical clustering of normalized expression values of the 21 fusion transcripts reveals that most of them have large variations in their expression profiles across the patient cohort, and no distinct sub-groups were identified (Supplementary Figure 1). Such divergent expression signatures may be indicative of tumor heterogeneity at the transcriptomic level.

**Table 1:** Nominated fusion transcripts

Gene symbols	ChromA^1^	ChromB^1^	Strand^2^	Distance between fusion partners (bp)	Fusion junction at splice site	Potential in-frame protein	No. of positive tumor samples	No. of positive benign samples	Fisher_P^3^	Exp_A (T)^4^	Exp_V (T)^5^	Exp_A (B)^4^	Exp_V (B)^5^	Wilcoxon_P^6^
*TMPRSS2-ERG*	21	21	--	2 802 774	Y	N	19	0	1.6E-06	6.20	5.36	0	*	*
*KLK4-KLK3*	19	19	+-	45 588	N	N	17	5	3.0E-03	1.38	2.89	0.59	2.6E-02	9.6E-03
*DNAJB1-TECR*	19	19	-+	333	N	N	31	8	6.7E-07	0.25	2.3E-02	0.14	5.8E-03	1.7E-02
*SMG5-TMEM79*	1	1	-+	110	N	N	20	6	1.0E-03	0.53	1.2E-01	0.29	4.6E-03	1.6E-02
*GOLM1-NAA35*	9	9	-+	3 848	N	N	42	30	8.2E-04	0.49	1.1E-01	0.29	2.5E-02	5.8E-04
*SMG5-PAQR6*	1	1	--	1 134	N	N	11	1	1.7E-03	0.21	2.4E-02	0.15	*	*
*C9orf163-SEC16A*	9	9	+-	5 806	N	N	34	11	8.2E-07	0.56	1.2E-01	0.27	2.3E-02	2.1E-03
*SLC45A3-ELK4*	1	1	--	25 889	Y	Y	14	5	1.8E-02	0.37	1.9E-01	0.20	4.3E-03	2.2E-02
**Novel fusion transcripts**														
*PXDN-AC144450.2*	2	2	--	6 468	N	N	27	1	4.2E-10	0.47	7.3E-01	0.14	*	*
*ACER3-B3GNT6*	11	11	++	7 544	N	N	19	1	2.1E-06	0.49	1.9E-01	0.13	*	*
*DSCC1-KB_1471A8.1*	8	8	-+	11 409	N	N	6	0	1.3E-02	0.07	6.5E-04	0	*	*
*ACSS1-APMAP*	20	20	--	13 253	N	Y	29	20	4.3E-02	0.31	3.1E-02	0.20	1.1E-02	4.6E-02
*SPON2-CTBP1*	4	4	--	2 486	N	N	14	2	8.2E-04	0.53	1.6E-01	0.12	1.9E-03	*
*SSBP2-CPNE4*	5	3	NA	NA	N	N	16	1	3.1E-05	1.42	4.39	1.08	*	*
*NSUN4-FAAH*	1	1	++	29 113	N	Y	6	0	1.3E-02	0.31	3.4E-02	0	*	*
*TMEM219-TAOK2*	16	16	++	589	Y	Y	10	2	2.5E-02	0.11	4.1E-03	0.06	9.5E-04	*
*RP11_321F6.1-SMAD6*	15	15	++	16 434	Y	N	5	0	2.8E-02	0.18	2.4E-02	0	*	*
*ZNF841-ZNF432*	21	21	--	15 613	N	Y	15	5	1.0E-02	0.15	4.8E-03	0.08	1.0E-03	1.6E-02
*ZNF551-ZNF776*	19	19	++	56 997	N	Y	9	1	7.4E-03	0.11	3.0E-03	0.07	*	*
*RP11_17A19.1-KCTD1*	18	18	--	30 220	Y	N	25	6	2.0E-05	0.58	3.2E-01	0.18	1.3E-02	7.2E-03
*FAM83H-RP11_429J17.6*	8	8	-+	339	N	N	15	0	1.4E-04	0.22	2.3E-02	0	*	*

^1^ Chromosome numbers for the A and B genes, *i.e*. upstream and downstream fusion partners

^2^ Which of the two DNA strands the fusion partner sequence corresponds to (+: positive strand; -: negative strand)

^3^ *P* value of Fisher's exact test for difference in positive fraction among tumor *vs*. benign samples

^4^ The average expression value for fusion transcripts in tumor and benign samples

^5^ The variance of expression estimate for the fusion transcripts in tumor and benign samples

^6^ *P* value of Wilcoxon rank test for differential expression between tumor and benign samples

* Too small sample size of benign tissues for statistics test

NA: Fusion partners are located on different chromosomes

### Novel fusion transcripts with high prevalence in prostate cancers

For the 21 identified fusion transcripts, the involved partner genes are mostly localized within 60 kb on the same chromosome, except *TMPRSS2-ERG* (intrachromosomal, distance approx. 3 Mb) and *SSBP2-SPNE4* (interchro mosomal). Thirteen of the fusion transcripts have not previously been described in prostate cancer (Table 1). The remaining eight are known fusion transcripts (*e.g. TMPRSS2-ERG*, *C9orf163-SEC16A*, *SMG5-TMEM79*, and *KLK4-KLK3*) [5, 21, 22], of which *TMPRSS2-ERG* is the most common, and was found to be positive in 19 (45%) of the 44 primary tumor samples (Table 1).

Among the novel fusion transcripts, eight are between pairs of classical protein-coding genes, whereas five include long intergenic non-coding RNA (lincRNA) as one of the fusion partners (see Table 1). Fusion transcripts such as *PXDN-AC144450.2* and *ACER3-B3GNT6* have an overrepresentation in tumors and underrepresentation in benign tissues with highly significant *p*-values (< 1E-05). In comparison, the fusion transcripts *RP11_17A19.1-KCTD1*, *ZNF841-ZNF432*, and *ACSS1-APMAP* show a common presence in normal samples as well, but they were found to be overexpressed in tumor *vs*. benign tissues (*p* < 0.05, Wilcoxon rank-test). To further confirm the high recurrence of these chimeras, an independent dataset of 50 additional primary tumors and seven prostate cancer cell lines were evaluated for the presence of any of the 13 novel fusion transcripts. We found that most of the transcripts could be found in similar proportions as in the 44 tumor-benign sample pairs (Supplementary Table 1). Only *RP11_17A19.1-KCTD1* and *RP11_321F6.1-SMAD6* show a slightly lower frequency from the 50 tumor samples. Moreover, four of the new nominated candidates were selected for RT-PCR validation, and three of them were successfully validated in five or all of the six prostate cancer cell lines (Supplementary Table 2). The breakpoints of fusion transcripts were verified by Sanger sequencing, and are identical to those found in RNA-seq data.

**Figure 2:**
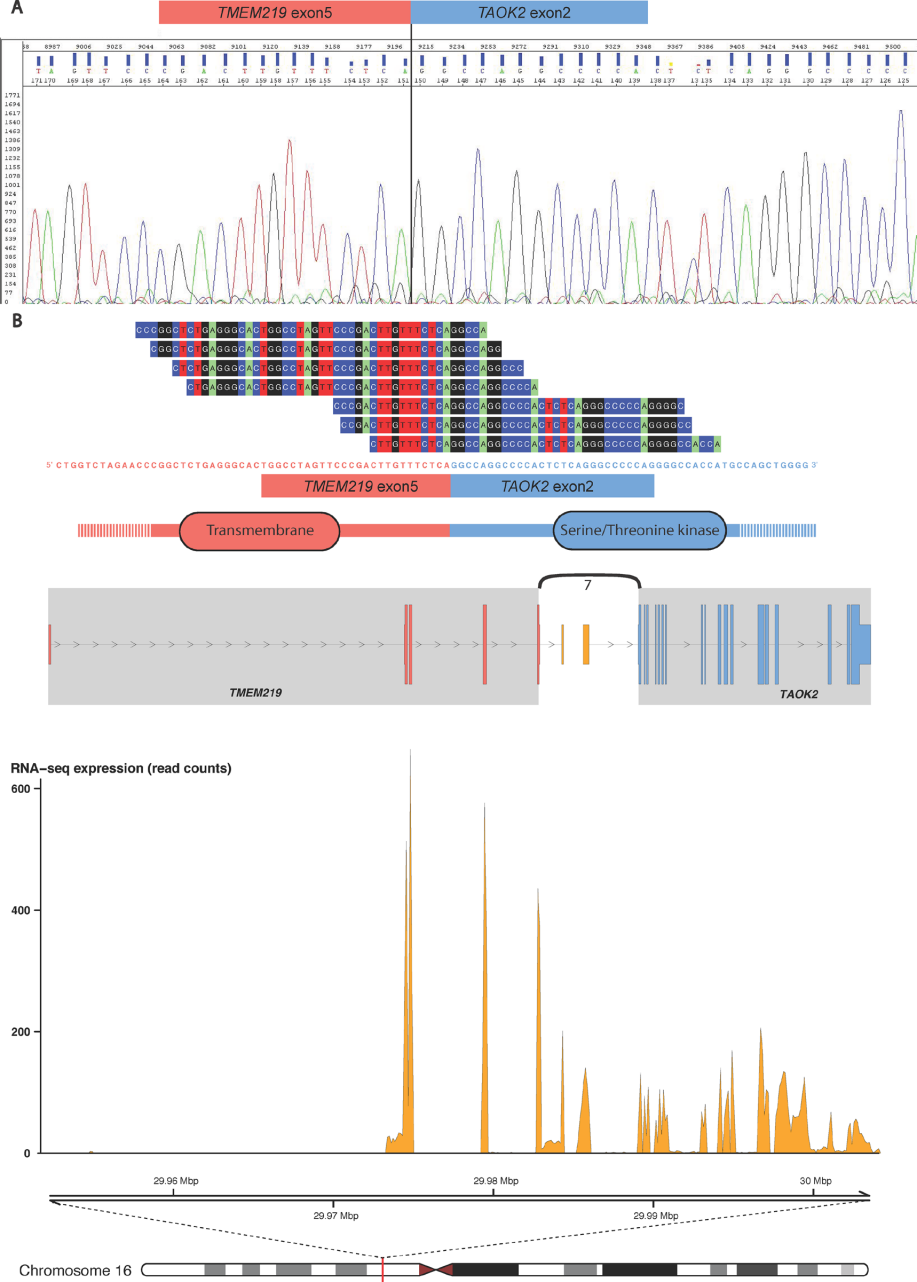
The *TMEM219-TAOK2* fusion transcript (**A**) The fusion transcript was detected from the VCaP, DU145, PC3, 22Rv1, NCI-H660 and LNCaP cell lines (see Supplementary Table 2), and the specific breakpoint was verified by Sanger sequencing. (**B**) Example data from the TCGA prostate tumor “TCGA-HC-7211-01A-11R-2118-07” shows seven split reads spanning the chimeric transcript breakpoint, from exon 5 of *TMEM219* (ENST00000414689) to exon 2 of *TAOK2* (ENST00000279394). The fusion transcript is predicted to include an in-frame open reading frame encoding a chimeric protein with the combination of transmembrane and serine/threonine kinase domains. The genomic view of the fusion event is from the top showing annotated exons of the fusion partner genes and the number of split reads supporting breakpoint (curved line), the RNA expression levels (read counts), and genomic coordinates for the fusion transcript in mega base pairs from the p-telomere of chromosome 16.

Next, we assessed whether the fusion transcripts can give rise to in-frame fusion proteins. In Table 1, we list that five of the novel fusion transcripts may translate into fusion proteins with truncated C-terminal of the header gene and an in-frame translation of the tail gene. One example, *NSUN4-FAAH*, may translate into a putative fusion protein composed of the Methyltransf_26 domain of *NSUN4* (exons 1 to 6) and the Amidase domain of *FAAH* (exons 2 to 15; Supplementary Figure 2). Two in-frame fusion transcripts involved partner genes encoding zinc finger family proteins, *ZNF841-ZNF432* and *ZNF551-ZNF776*, which might lead to coupling of two ZNF domains. Furthermore, the fusion transcript *TMEM219-TAOK2*, has intact splicing sites and reading frames of both fusion partners. The fusion protein may include parts encoded from the first five exons (240 amino acids) of *TMEM219* and exons 2 to 16 (1122 C-terminal amino acid residues) of *TAOK2*, which combines a transmembrane and a serine/threonine kinase (S_TKc) domain (see Figure 2).

In addition, we found that two fusion transcripts, *RP11_17A19.1-KCTD1* and *RP11_321F6.1-SMAD6*, are predicted not to encode fusion proteins. Instead, both combine a lincRNA gene as the upstream partner and a protein-coding gene as the downstream partner, using intact exon splicing sites from their fusion partner genes. We identified on average 20 and 5 split reads supporting the junctions (exon1–exon2 (*RP11_17A19.1-KCTD1*; Supplementary Figure 3) and exon3–exon2 (*RP11_321F6.1-SMAD6*; Supplementary Figure 4), respectively). The 3′ gene partner retains an intact (*KCTD1*) or truncated (*SMAD6*) coding sequence in the putative fusion proteins. In particular, the fusion transcript *RP11_17A19.1-KCTD1* is highly prevalent, being detected in 25 of the 44 (56%) and 14 of the 50 (28%) prostate tumors. This fusion transcript was as well detectable in six of the 44 benign prostate samples. Importantly, its expression level in tumors is significantly higher than that in benign prostates (*p* = 7.2E-03, Wilcoxon rank-test; Table 1).

### Expression of fusion transcripts and their 3′ fusion partner genes

**Figure 3:**
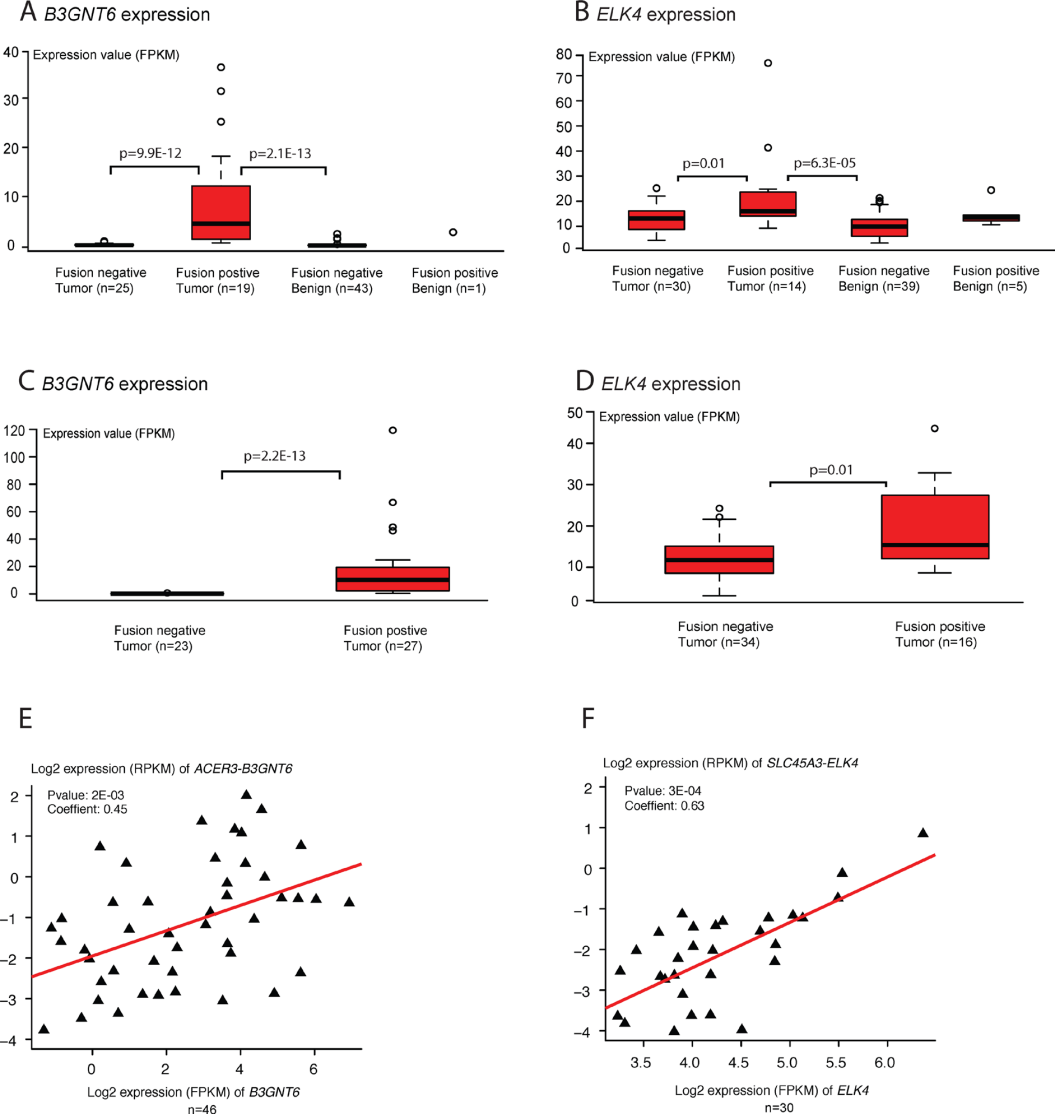
Differential expression of 3′ fusion partner genes (**A**) and (**B**) show the expression of the 3′ partner genes, *B3GNT6* and *ELK4*, of the fusions *ACER3-B3GNT6* and *SLC45A3-ELK4* in 44 pairs of tumor and benign prostate samples. (**C**) and (**D**) show the expression of 3′ partner genes *B3GNT6* and *ELK4* in 50 additional prostate tumors. (**E**) and (**F**) show the correlation between the expression of *ACER3-B3GNT6* and *SLC45A3-ELK4* and their respective 3′ partner genes, *B3GNT6* and *ELK4*. X and Y axes represent log2 transformed expression values (FPKM, fragments per kilobase of transcript per million mapped reads; RPKM, reads per kilobase of transcript per million mapped reads).

We compared the expression levels of fusion transcripts to these of the fusion partner genes, in both tumor and benign prostate samples. Our results show that most fusion partner genes are expressed at a moderate or low level. Of interest, several of the 3′ fusion partners are differentially expressed between samples with and without detection of the fusion transcripts. For instance, *B3GNT6* is highly expressed in the *ACER3-B3GNT6* positive tumor samples, and almost absent in both the fusion-negative tumor samples (*p* = 9.9E-12) and benign samples (*p* = 2.1E-13, Wilcoxon rank-test; Figure 3A). Similarly, the fusion partner *ELK4* is up-regulated in tumor samples expressing the *SLC45A3-ELK4* fusion in comparison with fusion-negative tumor samples (*p* = 0.01) and benign samples (*p* = 6.3E-05, Wilcoxon rank-test; Figure 3B). The same pattern was found from the 50 additional tumor samples (Figure 3C and 3D). Moreover, the expression of fusion transcripts *ACER3-B3GNT6* and *SLC45A3-ELK4* were found positively correlated to the expression of their downstream partners (*p* = 2E-03 and *p* = 3E-04, Spearman correlation test; Figure 3E and 3F). In differential exon-level expression analysis, exon 1 (only in wild type) of *ELK4* shows a higher expression in fusion negative tumors than in fusion positive tumors (Supplementary Figure 7A). In comparison, exons 2–6 (sum of fusion and wild type) of *ELK4* are stronger expressed in fusion positive tumors (Supplementary Figure 7A). Quantitative RT-PCR analyses confirm that the expression of *ELK4* exon 2 (sum of fusion and wildtype) is significantly higher than that of exon 1 (wildtype) in 39 prostate tumor samples (*p* = 3.1E-10, Wilcoxon rank-test; Supplementary Figure 8A). And the expression of the fusion transcript shows a significant positive correlation to that of *ELK4* exon 2 (*r* = 0.83 and *p* = 5.6E-11, Spearman correlation test; Supplementary Figure 8B). There was no correlation between the expression of *ELK4* exon 2 and *ELK4* exon 1 (*r* = −0.1 and *p* = 0.43, Spearman correlation test; Supplementary Figure 8C). These results support that the induced expression of the downstream fusion partner is driven by the expression of the fusion transcript, which again most likely is mediated by RNA polymerase read-through from the upstream partner gene. It further indicates that the formation of transcription-induced fusion transcripts may be accompanied with induced expression of the downstream fusion partner.

## DISCUSSION

In this study, we carried out a comprehensive survey of fusion transcripts in RNA-sequencing data from 44 pairs of prostate cancer and benign tissues being sequenced by the TCGA consortium [3]. Thirteen novel recurrent fusion transcripts were nominated using stringent filtering criteria. In general, these chimeras are characterized by having partner genes which are localized less than 60 kb apart and are transcribed from the same strand (10/13 gene pairs). Thus, they are likely produced by transcription-induced fusion events, and not genomic rearrangements. As several of the fusion transcripts were also present in the benign prostate samples (Table 1), the selection of sufficient normal samples as control is therefore of key importance to distinguish potential driver fusions from passengers. If the fusion transcripts are only detected in 1–2 normal samples (like *ACER3-B3GNT6* and *PXDN-AC144450.2*), we suggest that they are probably involved in the cancer development. Their rare presence in benign prostate tissues may represent a ”field effect” within the normal epithelium preceding histological change [27], or a possible contamination by marginal tumor cells in the normal tissue during sampling. Thus, a further validation from more patient samples and cell lines would be helpful to clarify the feature of such fusion event in normal tissue. On the other hand, several fusion transcripts (*e.g.SLC45A3-ELK4* and *C9orf163-SEC16A*) show a common presence in normal samples (Table 1). The comparison of the expression level of fusion transcripts in tumors *vs*. benign tissues enabled us to nominate the ones overexpressed in cancers as likely involved in cancer-related pathological processes. Regardless of the fusion transcripts being detected in benign tissues, their high prevalence in the 94 investigated tumor samples indicate that the expression of these fusion transcripts may be relevant to pathological processes and malignant transformation. Our study expands the scope of classical fusion genes derived by chromosome rearrangement to include more potential driver fusions mediated at the transcriptome level.

Two important mechanisms have been proposed to mediate the formation of transcription-induced fusion genes, RNA polymerase read-through and *trans*-splicing (see review [28]). Although both can produce chimeras by splicing events, the read-through scenario may be coupled with an induced expression level of the 3′ gene partner, since the fusion transcript is transcribed from the promoter of the 5′ gene. In this study, up-regulation of 3′ gene partners were seen for the fusion transcripts *SLC45A3-ELK4*, *ACER3-B3GNT6*, *ZNF841-ZNF432* and *PXDN-AC144450.2*, when comparing fusion-positive to fusion-negative tumor samples (Figure 3 and Supplementary Figure 6). A significant differential usage of exon was shown in 3′ partner gene *ELK4*, and less so for the 3′ partner gene *ZNF432* (Supplementary Figure 7A and 7B). Quantitative RT-PCR analyses of exons up- and downstream of the breakpoint in the fusion partner *ELK4* (exon1 and 2), support that the expression of fusion transcript contributes significantly to the up-regulation of the 3′ gene partner *ELK4* (Supplementary Figure 8). For the 3′ fusion partners *B3GNT6* and *AC144450.2* this was not seen (Supplementary Figure 7C and S7D), and we cannot rule out other mechanisms than RNA polymerase read-through.

In addition, *B3GNT6* was also found as 3′ gene partner in the fusion *SOD2-B3GNT6* (data not shown), this fusion transcript was only detected in one tumor sample. Its low prevalence suggests that the increased expression of *B3GNT6* is mainly mediated by the control of the *ACER3* promoter. In addition to the *SLC45A3-ELK4*, we found three other fusion transcripts involving *ELK4* (data not shown). However, in all these, *ELK4* was involved as the 5′ partner gene. Overall, our findings indicate that each of the four fusions is transcribed as a single transcript, generated by RNA polymerase read-through of both partner genes, and their presences contribute to a higher expression of the 3′ partner genes. However, the formation of fusion transcripts by a read-through mechanism is only one possible explanation for the increased expression of 3′ partner genes. Since the transcription of parental genes is not broken off by the read-through, also other factors may contribute to the up-regulation of partner genes.

Despite the high recurrence of 13 novel fusion transcripts in prostate tumors, their biological roles are not obvious. As shown in Supplementary Table 3, fusion formation may have impact on several cellular pathways, such as the apoptotic process (*TMEM219-TAOK2*), fatty acid metabolism (*NSUN4-FAAH*), glycoprotein biosynthesis (*ACER3-B3GNT6*), membrane trafficking (*SSBP2-CPNE4*), cell adhesion (*SPON2-CTBP1*), and transcript regulation (*ZNF841-ZNF432* and *ZNF551-ZNF776*). The fusion transcripts can be translated into in-frame fusion proteins with intact or truncated coding region of the fusion partner genes. They may as well have new 3′-untranslated regions, which could alter the translational efficiency.

Three fusion transcripts, *TMEM219-TAOK2*, *NSUN4-FAAH*, and *ACER3-B3GNT6*, show a functional implication to prostate cancer development and progression. Firstly, *TMEM219* is a transmembrane protein located in the plasma membrane, whereas *TAOK2* (also named “prostate-derived sterile 20-like kinase 1”) is a serine/threonine protein kinase that is usually expressed in the nucleus. Both proteins are involved in the apoptotic process and are related to development of prostate cancer [29, 30], in particular *TAOK2* is critical in activation of MAPKK pathway and regulation of cell growth [31]. The putative chimeric protein combines an intact transmembrane domain and a serine/threonine kinase domain (Figure 2), and may induce the kinase activity outside of the nucleus. For the fusion *NSUN4-FAAH* (Supplementary Figure 2), *NSUN4* is an rRNA methyltransferase involved in mitochondrial ribosome assembly and FAAH is a fatty acid amide hydrolase that participates in fatty acid catabolic process [32, 33]. The putative NSUN4-FAAH chimeric protein shows a domain rearrangement which integrates the core catalytic function of the two partners. Although no studies have indicated a possible role in tumorigenesis for the two genes, FAAH has previously been reported to be highly expressed in prostate tissue [34, 35]. As discussed, the presence of fusion *ACER3-B3GNT6* (Figure 3 and Supplementary Figure 5) is significantly associated with an up-regulation of *B3GNT6* expression level. *B3GNT6* is short for β-1,3-N-acetylglucosaminy transferase 6 and has an important role in the synthesis of mucin-type O-glycans in the Golgi apparatus. Increased *B3GNT6* expression has previously been reported in prostate cancer, and it may be involved in the metastatic capacity of cancer cells [36]. In this study, a high expression of *B3GNT6* was found in 19 of 44 (43%) tumor samples.

We found that lincRNAs are commonly involved in the formation of fusion transcripts. As shown in Table 1, five of the 13 nominated fusion candidates included lincRNAs (two as upstream partner and three as downstream partner). One previously characterized lincRNA (*RP11_321F6.1*, also known as *PCAT18*), is shown to be a regulator of prostate cancer cell proliferation, invasion and migration [37]. Another fusion transcript frequently expressed in prostate tumors involves the lincRNA *AC144450.2* with unknown function. Since lincRNAs regulate gene expression in cancer cells and may play oncogenic or tumor suppressive roles [38], the investigation of biological implication of lincRNA fusion transcripts may provide new insights into tumorigenesis at the transcription regulatory level, and can be a source of cancer biomarker and therapeutic targets.

Notably, no partner genes of the novel fusion transcripts belong to the ETS transcription factor gene family. Further, most of the novel fusions are expressed in less than 50% of the tumor samples and show a scattered distribution across the cohorts (Supplementary Figure 1). This supports a strong inter-tumor heterogeneity, which is also clearly indicated in earlier studies of DNA copy numbers, somatic mutations and epigenetic alterations [2, 3, 24, 39]. In conclusion, we have here identified a set of novel fusion transcripts being overexpressed in prostate cancer. These fusion transcripts form an additional layer of cellular complexity in prostate cancers with yet unknown implications for the development and management of this disease.

## MATERIALS AND METHODS

### Data preparation

Raw RNA sequencing data in fastq format from TCGA (44 pairs primary tumors and benign tissues; 50 unpaired primary tumors) were downloaded from Cancer Genome Hub (CGHub, https://cghub.uscs.edu, now changed to GDC data portal: https://gdc-portal.nci.nih.gov). RNA sequencing data in BAM format for seven prostate cancer cell lines (Cancer Cell Line Encyclopedia) were obtained from CGHub repository and aligned reads were extracted using bam2fastq. The summary of RNA-seq data from the TCGA and Cancer Cell Line Encyclopedia is listed in Supplementary Table 4. Clinical data, *i.e*. pre- and post-operation values for prostate specific antigen (PSA) and Gleason scores, were downloaded from the FTP server of TCGA.

### Fusion detection and filtering

We used the deFuse software (version 0.6.1), with hg19 human reference genome sequence and Ensembl release 69 annotation database, to predict fusion transcripts from the RNA sequencing data for both primary tumors and benign tissues [40]. Briefly, deFuse detected fusion transcripts through the identification of discordant read pairs and junction split reads. The preliminary deFuse output of fusion RNA predictions were generated according to the following criteria: 1) at least five discordant read pairs, 2) at least one junction split read and 3) deFuse classifier probability no less than 0.05. To better evaluate the fusions that showed presence in both tumor and benign samples, we approximately quantified the expression of fusion transcripts by calculating the number of split reads mapped to breakpoint region (*i.e*. reads per kilobase of transcript per million mapped reads; measured as RPKM value).

Several specific filtering steps were further applied for reducing the number of false positives based on statistics data mining: 1) fusions had to be present in at least five tumor samples; 2) the number of fusions was enriched in tumors compared to that in benign prostates (*p* < 0.05; Fisher's exact test); 3) if fusions present in more than two benign prostates, they were retained only when the expression level was higher in tumor as compared to normal (*p* < 0.05; Wilcoxon signed rank test); 4) the ratio of multi-mapping spanning reads to total spanning reads should be less than 0.6 (*i.e*. the control of possible ambiguous mapping on both sides of the predicted breakpoints); 5) both gene partners of fusion transcripts had to be either protein-coding, lincRNA or miRNA genes, and putative fusions between overlapping genes were discarded. All fusion candidates nominated by these procedures were manually reviewed in the UCSC Genome Browser by BLAT (http://genome.ucsc.edu/cgi-bin/hgBlat?command=start) to double-check the sequence specificity in breakpoint region.

### Functional annotation

Annotations of the gene partners involved in the formation of fusion candidates were assigned by Gene ontology (GO) terms in three major categories: Molecular function (MF), biological process (BP) and cellular component (CC) (http://www.geneontology.org/). The terms with evidence inferred from electronic annotation (IEA) were discarded in this study. Putative chimeric proteins that hold in-frame coding sequences were searched for structural domains and motifs against Pfam and SMART databases [41, 42]. A domain was considered present in the chimeric protein if it had an *e*-value <= 1E-6. Particular attention was paid to breakpoints that occurred outside of structural domains.

### Expression profile analysis

RNA-seq raw reads were mapped onto the hg19 genome and exon-exon junction by splice-aware aligner TopHat 2.0.11 [43]. Gene expression profile for each sample was calculated using Cufflinks 2.2.0 based on the Ensembl gene annotation (release 69) [44], and the expression were quantified in fragments per kilobase of gene per million mapped reads (FPKM). The differential expression level of fusion transcripts (*i*) between 44 pair tumor-benign samples (*j*) was normalized using: $\mathrm{valu}e_{\mathrm{ij}}^{'}=\left(\frac{\mathrm{Tex}p_{\mathrm{ij}}-\mathrm{Nex}p_{\mathrm{ij}}}{\underset{j=1.44}{\text{max}}\left|\mathrm{Tex}p_{\mathrm{ij}}-\mathrm{Nex}p_{\mathrm{ij}}\right|}\right)$, where *value*_ij_^'^ is the normalized differential expression value, *Texp*_ij_ and *Nexp*_ij_ represent the expression levels of fusion transcript *i* in tumor and matched normal tissue *j*. Hierarchical clustering analysis using Pearson correlation as distance matrix was done in R 3.1.2. Fusion breakpoints, the transcript annotation of gene partners and sequencing coverage were visualized using R package chimeraviz (submission to Bioconductor; https://www.bioconductor.org/packages/devel/bioc/html/chimeraviz.html). Differential exon usage analyses were performed by DEXSeq package after counting reads per exon using “dexseq_count.py” script [45]. Samples were normalized using function “estimateSizeFactor” and variability was estimated using function “estimateDispersons”. Statistical testing of differential exon usage between fusion positive and negative conditions was done using function “testForDEU” (*q*-value < 0.1 was considered as significant).

### RT-PCR and validation of fusion transcripts on Prostate cancer cell lines

RNA was isolated from six prostate cancer cell lines (DU145, PC3, VCaP, 22Rv1, NCI-H660 and LNCaP) using AllPrep DNA/RNA/miRNA Universal kit (Qiagen, Hilden, Germany), and cDNA synthesis was performed using the High Capacity cDNA Reverse Transcription kit (Applied Biosystems by ThermoFisher Scientific (AB), MA, USA). Validation was performed by RT-PCR across the predicted breakpoint regions. Reactions creating visible bands on an agarose gel were sequenced directly (AB 3730 DNA Analyzer, AB) to validate further the presence of the fusion transcripts (primers can be found in Supplementary Table 5; Supplementary Figure 9).

### Quantitative real-time reverse transcriptase PCR

Included in the analyses were RNAs from 39 fresh frozen prostate cancer samples from prostatectomies undertaken at the Oslo University Hospital-Radium-hospitalet during 2010–2012. The RNA was isolated and cDNA synthesized as described above for the cell lines. Expressions of the fusion transcript *SLC45A3-ELK4* and of exons up- and downstream of the breakpoint in *ELK4* (exon 1 and exon 2) were analyzed using three TaqMan assays (AB). The assays measuring the individual exons in *ELK4* were as designed by AB (exon 1, Hs00360812; exon 2, Hs00360813_m1). The assay specifically measuring the chimeric fusion breakpoint (primers and probe in Supplementary Table 5) was custom designed with Primer Express 3.0 based on parameters given by the Primer Express software (AB). For each reaction, 10 ng cDNA was used. For the predesigned assays, 5 μl TaqMan Universal Master Mix II with UNG (2X), 0.5 μl assay and 1.5 μl RNase free water were added to a total volume per well of 10 μl. For the custom designed assay 0.09 μl of each primer (100 uM), 0.2 μl probe (10 uM) and 1.62 μl RNase free water were combined to a total volume of 10 μl. The reactions were incubated at 50°C for 2 minutes followed by 95°C for 10 minutes and 40 cycles of 95°C for 15 seconds 60 for 1 minute. Fluorescence was measured on an ABI 7900HT Fast Real-time PCR System (AB). All assays were run in triplicates on ABI 7900HT Fast Real-time PCR System, and expression levels were reported as the median cycle threshold (C_T_) of the triplicates.

## SUPPLEMENTARY MATERIALS FIGURES AND TABLES








