# Supplementary material for: Novel transcription-induced fusion RNAs in prostate cancer
Source: Oncotarget. 2017 Apr 13;8(30):49133–43. doi: 10.18632/oncotarget.17099 (PMC5564755; doi:10.18632/oncotarget.17099)
Supplement: Supplementary file 1 [file oncotarget-08-49133-s001.pdf]

Novel transcription-induced fusion RNAs in prostate cancer

Supplementary Materials

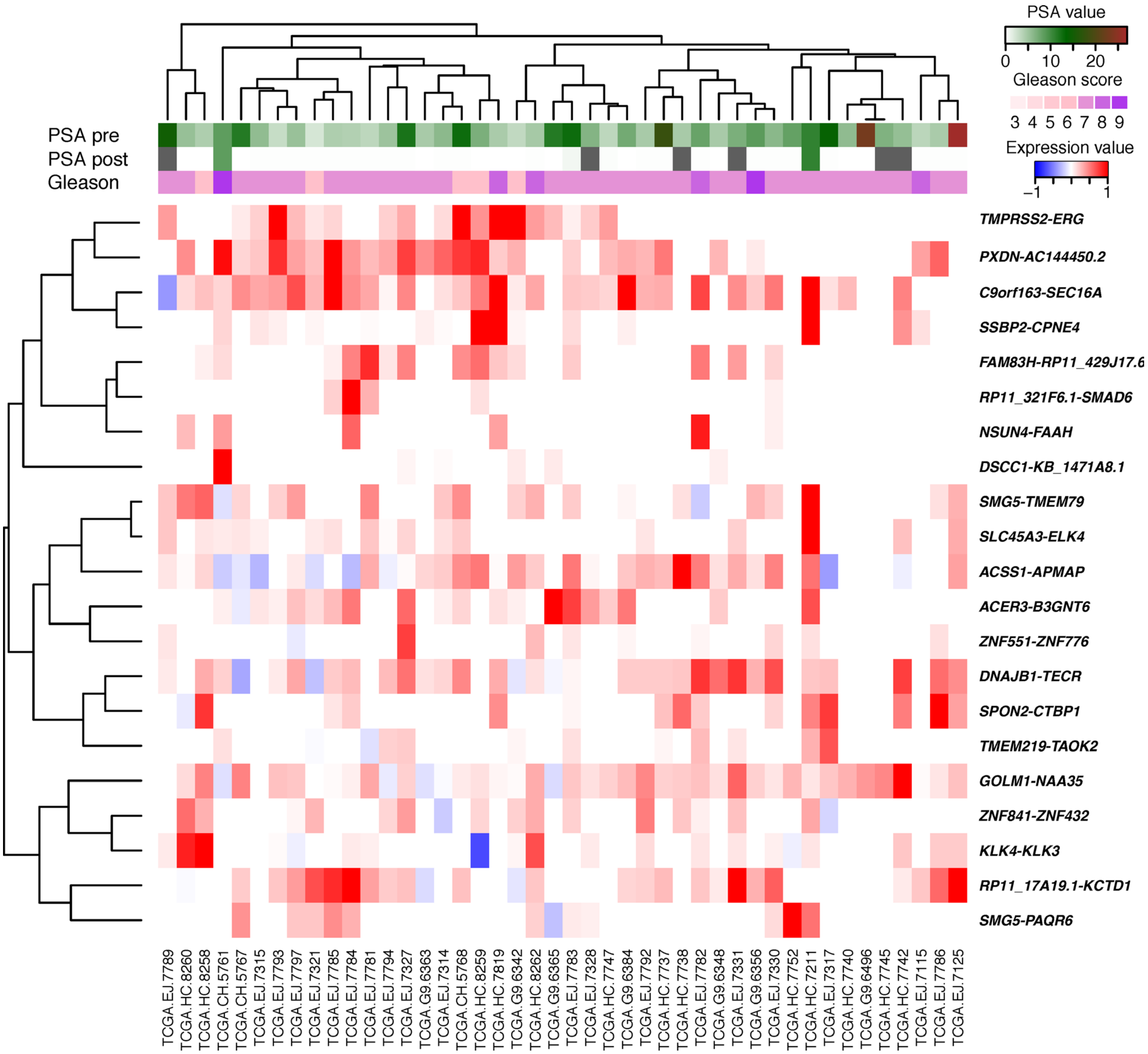

Supplementary Figure 1: Fusion transcript landscape in prostate cancers. The heatmap represents the unsupervised hierarchical clustering analysis (using correlation coefficient as distance matrix) of expression levels of the 21 identified fusion transcripts. Rows represent the different fusion transcripts, and columns represent the individual prostate cancers from a cohort of 44 patients. The blue-to-red color gradient in the cell shows relative expression value of fusion transcript in the tumor as compared to that in the corresponding benign prostate. Samples are annotated with PSA value measured before (PSA pre) and after (PSA post) operation, and total Gleason score. Grey color indicates no PSA value available in follow-up after operation.

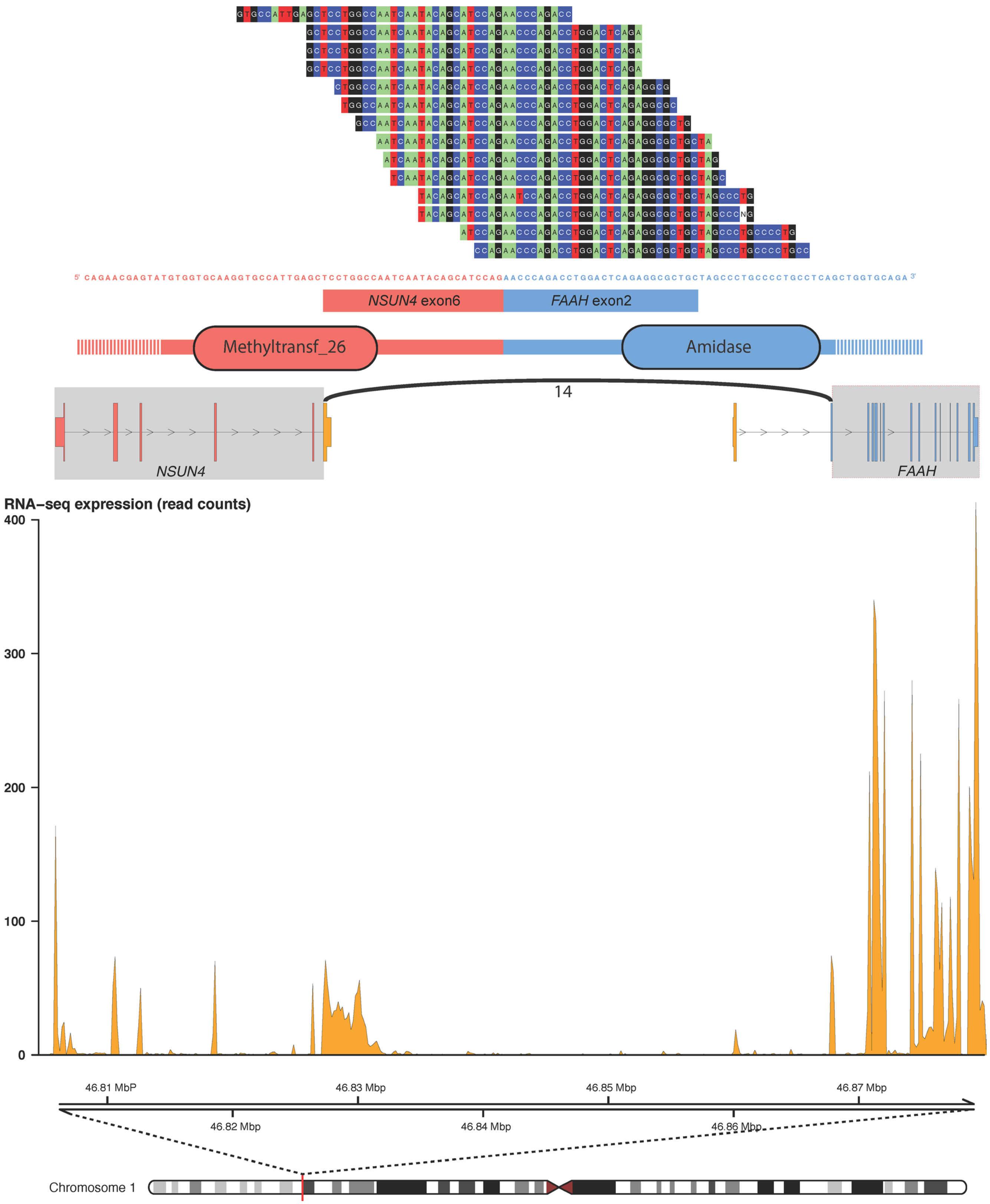

Supplementary Figure 2: The *NSUN4-FAAH* fusion transcript. Example data from the TCGA prostate tumor "TCGA-EJ-7784-01A-11R-2118-07" shows 14 split reads crossing the chimeric transcript breakpoint, from exon 6 of *NSUN4* (ENST00000474844) to exon 2 of *FAAH* (ENST00000243167). The fusion transcript is predicted to include an in-frame ORF encoding a chimeric protein with the combination of Methyltransf_26 and Amidase domains. The genomic view of the fusion event is from the top showing annotated exons of the fusion partner genes and the number of split reads supporting the breakpoint (curved line), the RNA expression levels (read counts), and the genomic coordinates for the fusion transcript in mega basepairs from the p-telomere of chromosome 1.

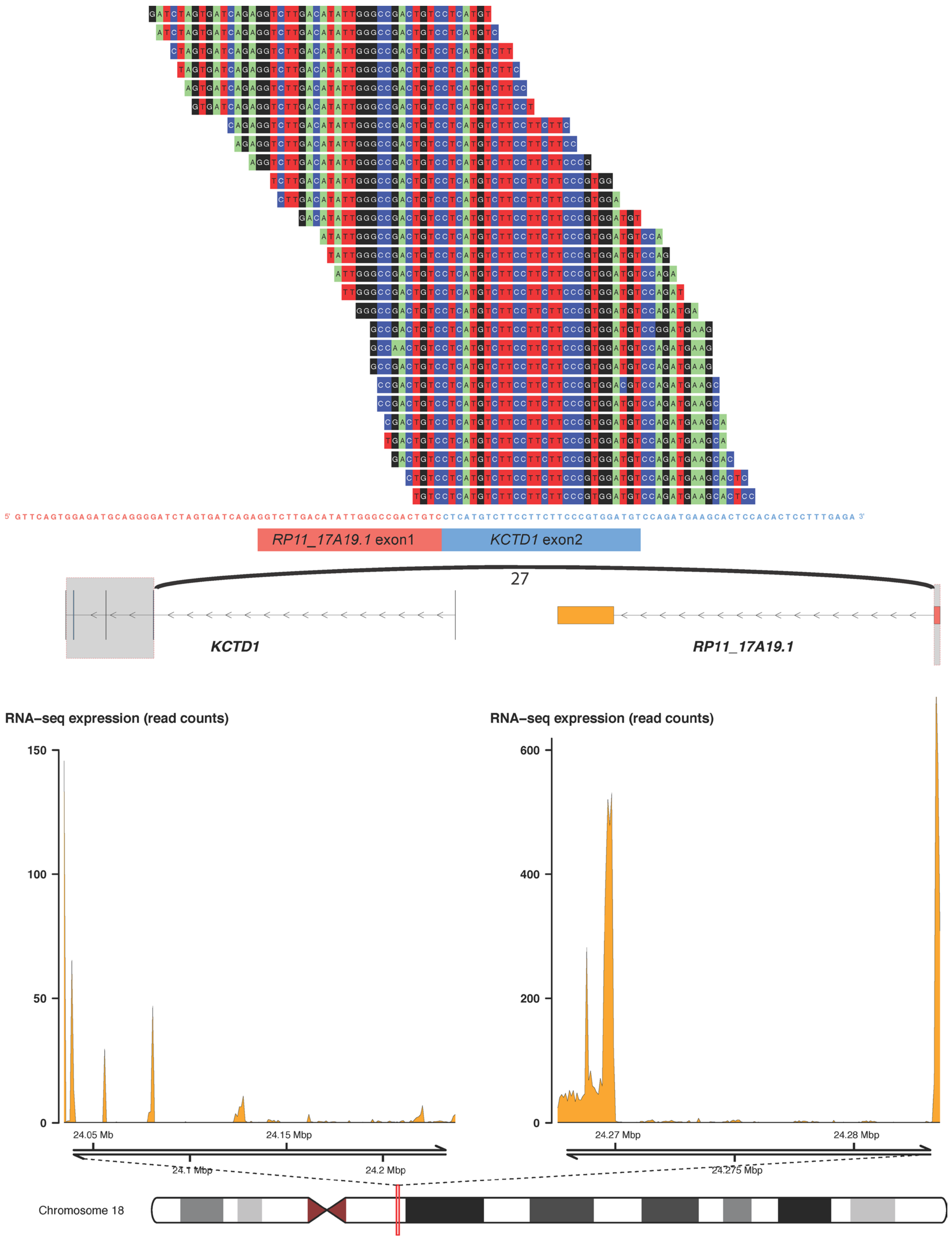

Supplementary Figure 3: The *RP11_17A19.1-KCTD1* fusion transcript. Example data from the TCGA prostate tumor "TCGA-EJ-7125-01A-11R-1965-07" shows 27 split reads crossing the chimeric transcript breakpoint, from exon 1 of *RP11_17A19.1* (ENST00000579458) to exon 2 of *KCTD1* (ENST00000580191). The genomic view of the fusion event is from the top showing annotated exons of the fusion partner genes and the number of split reads supporting the breakpoint (curved line), the RNA expression levels (read counts), and the genomic coordinates for the fusion transcript in mega basepairs from the p-telomere of chromosome 18.

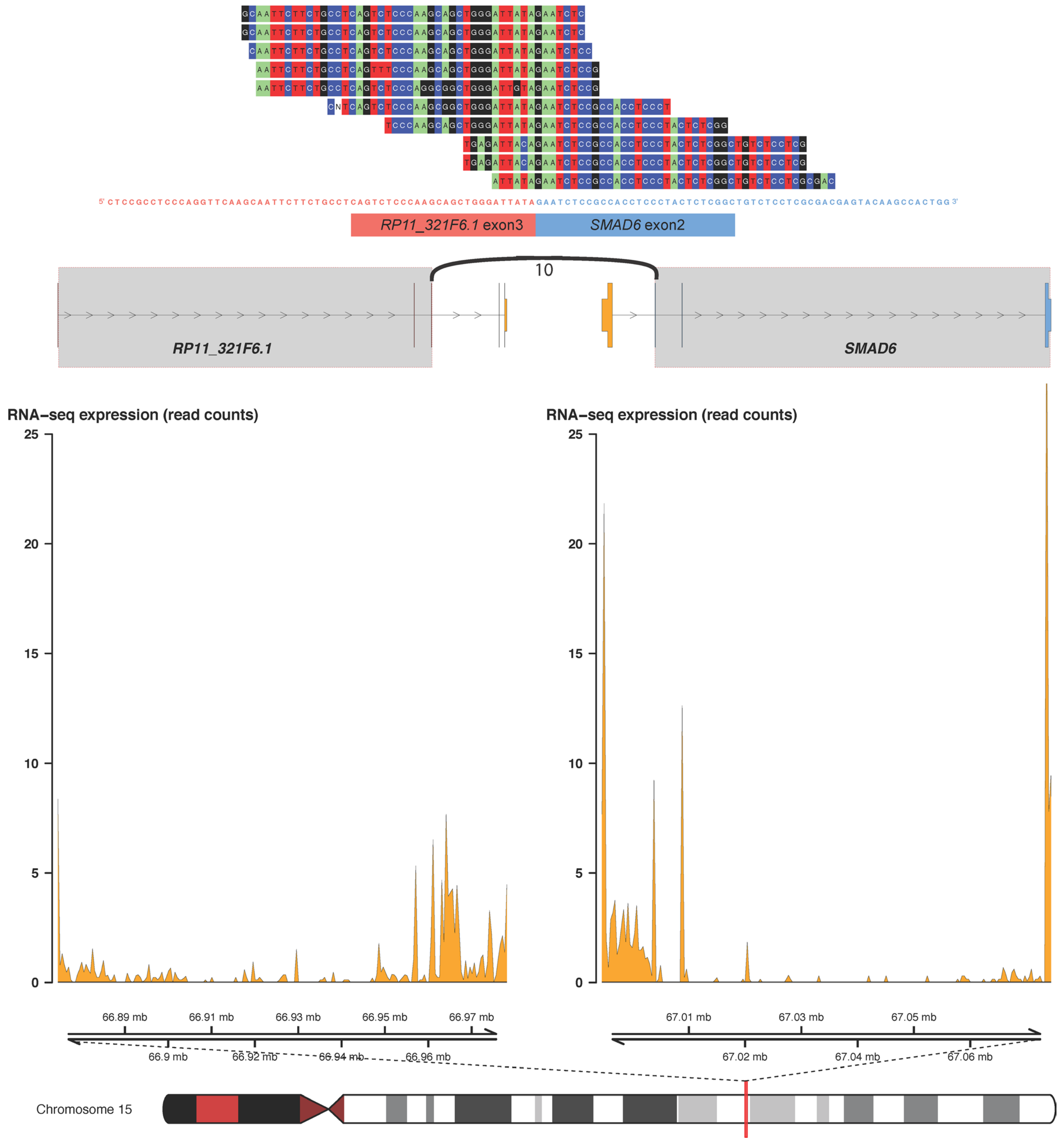

Supplementary Figure 4: The *RP11_321F6.1-SMAD6* fusion transcript. Example data from the TCGA prostate tumor "TCGA-EJ-7784-01A-11R-2118-07" shows 10 split reads crossing the chimeric transcript breakpoint, from exon 3 of *RP11_321F6.1* (ENST00000558797) to exon 2 of *SMAD6* (ENST00000288840). The genomic view of the fusion event is from the top showing annotated exons of the fusion partner genes and the number of split reads supporting the breakpoint (curved line), the RNA expression levels (read counts), and the genomic coordinates for the fusion transcript in mega basepairs from the p-telomere of chromosome 15.

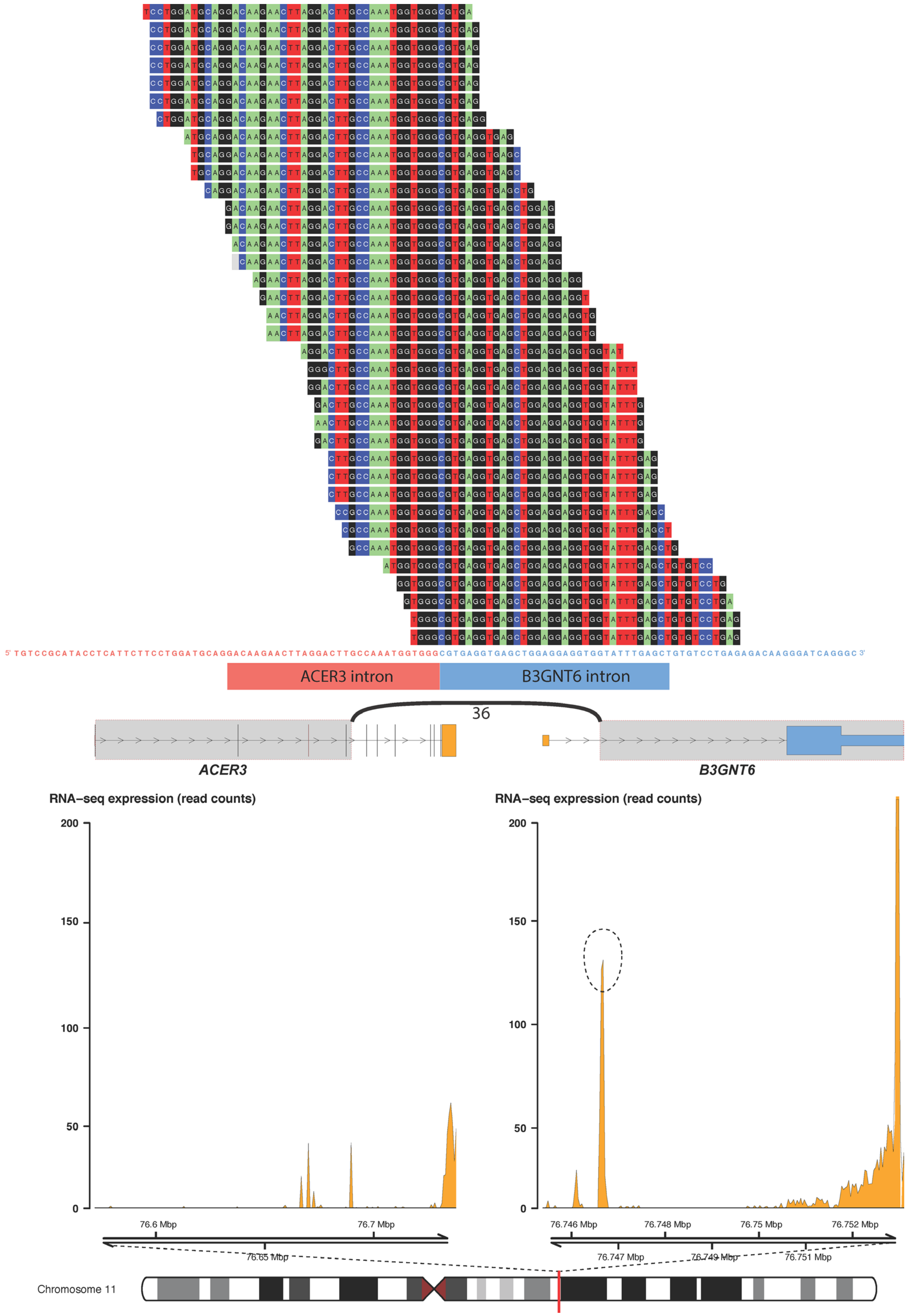

Supplementary Figure 5: The *ACER3-B3GNT6* fusion transcript. Example data from the TCGA prostate tumor "TCGA-G9-6365-01A-11R-1789-07" shows 36 split reads crossing the chimeric transcript breakpoint, from sequences within intron 4 of *ACER3* (ENST00000532485) and intron 1 of *B3GNT6* (ENST00000533140). The genomic view of the fusion event is from the top showing annotated exons of the fusion partner genes and the number of split reads supporting the breakpoint (curved line), the RNA expression levels (read counts), and the genomic coordinates for the fusion transcript in mega basepairs from the p-telomere of chromosome 11.

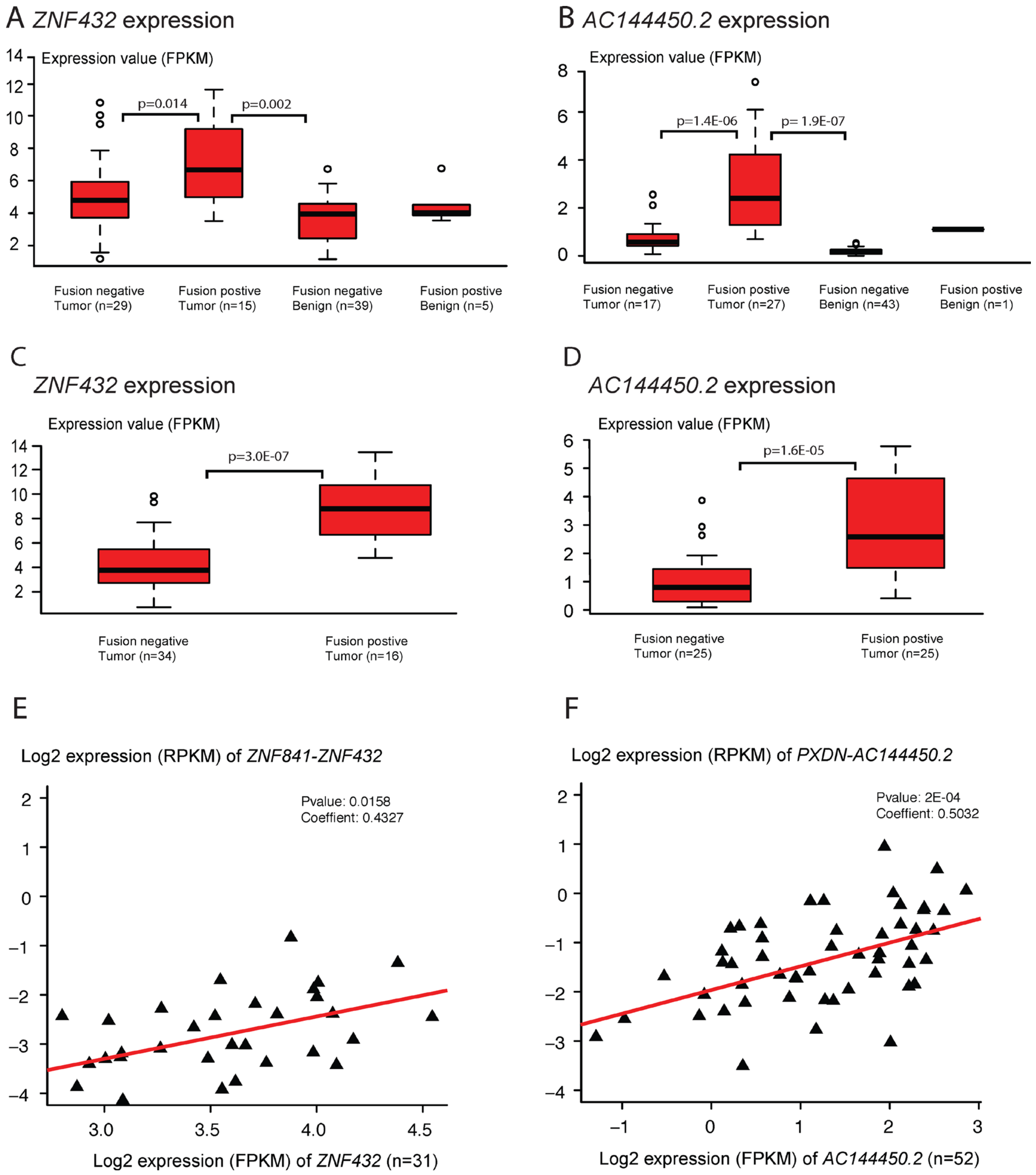

Supplementary Figure 6: Differential expression of 3′ fusion partner genes. (**A**) and (**B**) show the expression levels of the 3' partner genes *ZNF432* and *AC144450.2* of the fusion transcripts *ZNF841-ZNF432* and *PXDN-AC144450.2* in 44 pairs of tumor and benign prostate samples. (**C**) and (**D**) show the expression levels of the 3′ partner genes *ZNF432* and *AC144450.2 i*n 50 additional prostate tumors. (**E**) and (**F**) show the correlation between expression of fusion *ZNF841-ZNF432* and *PXDN-AC144450.2* and their 3′ partner genes *ZNF432* and *AC144450.2* in the respective fusion positive tumors. X and Y axes represent log2 transformed expression values (FPKM, fragments per kilobase of transcript per million mapped reads; RPKM, reads per kilobase of transcript per million mapped reads).

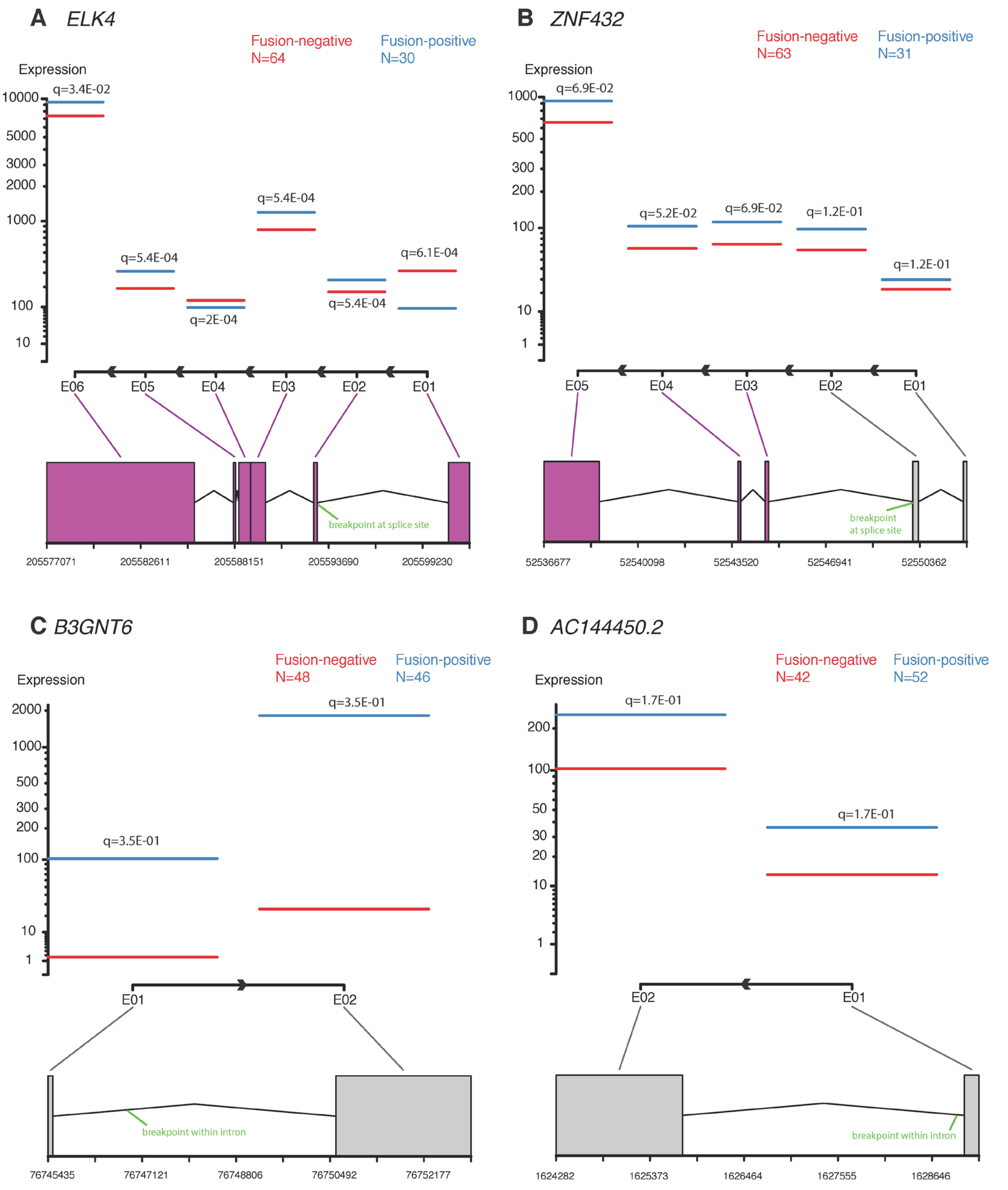

Supplementary Figure 7: Differential exon usage analysis of 3' fusion partner genes. (**A**) *ELK4* (ENSG00000158711), (**B**) *ZNF432* (ENSG00000256087), (**C**) *B3GNT6* (ENSG00000198488), and (**D**) *AC144450.2* (ENSG00000203635) show differential exon usage between fusion positive and negative tumors in prostate cancers. The breakpoint site of each 3′ fusion partner gene is marked with a green line and font. The exon expression levels are represented by normalized read counts. In the exon representation below, a significant differential exon usage (q-value < 0.1) is indicated with purple fill color.

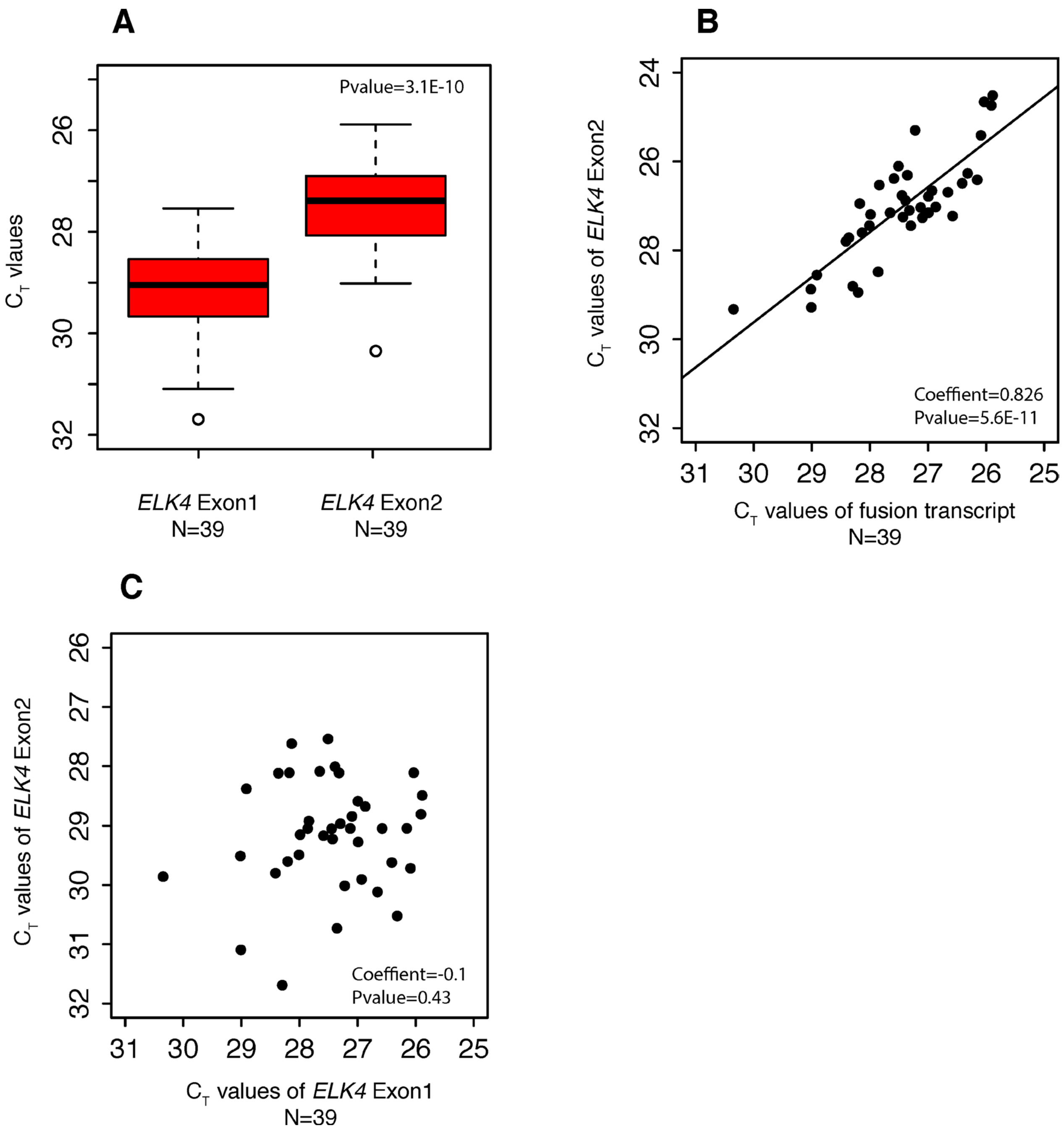

Supplementary Figure 8: Quantitative RT-PCR analyses of the expression of *ELK4* exon1, exon2 and the *SLC45A3-ELK4* fusion transcript. The expression level was quantified by cycle threshold value (C_T), with higher expression corresponding to lower CT values. The expression of exons 1 and 2 represent *ELK4* wildtype and *ELK4* wildtype + *SLC45A3-ELK4* fusion transcript, respectively. (**A**) Comparison of the expression of *ELK4* exon 1 versus exon 2; *P*-value calculated by Wilcoxon rank-test. (**B**) and (**C**) The expression correlation of *ELK4* exon 2 to *SLC45A3-ELK4* fusion transcript (B) and *ELK4* exon 1 (C); P-value calculated by Spearman correlation test.

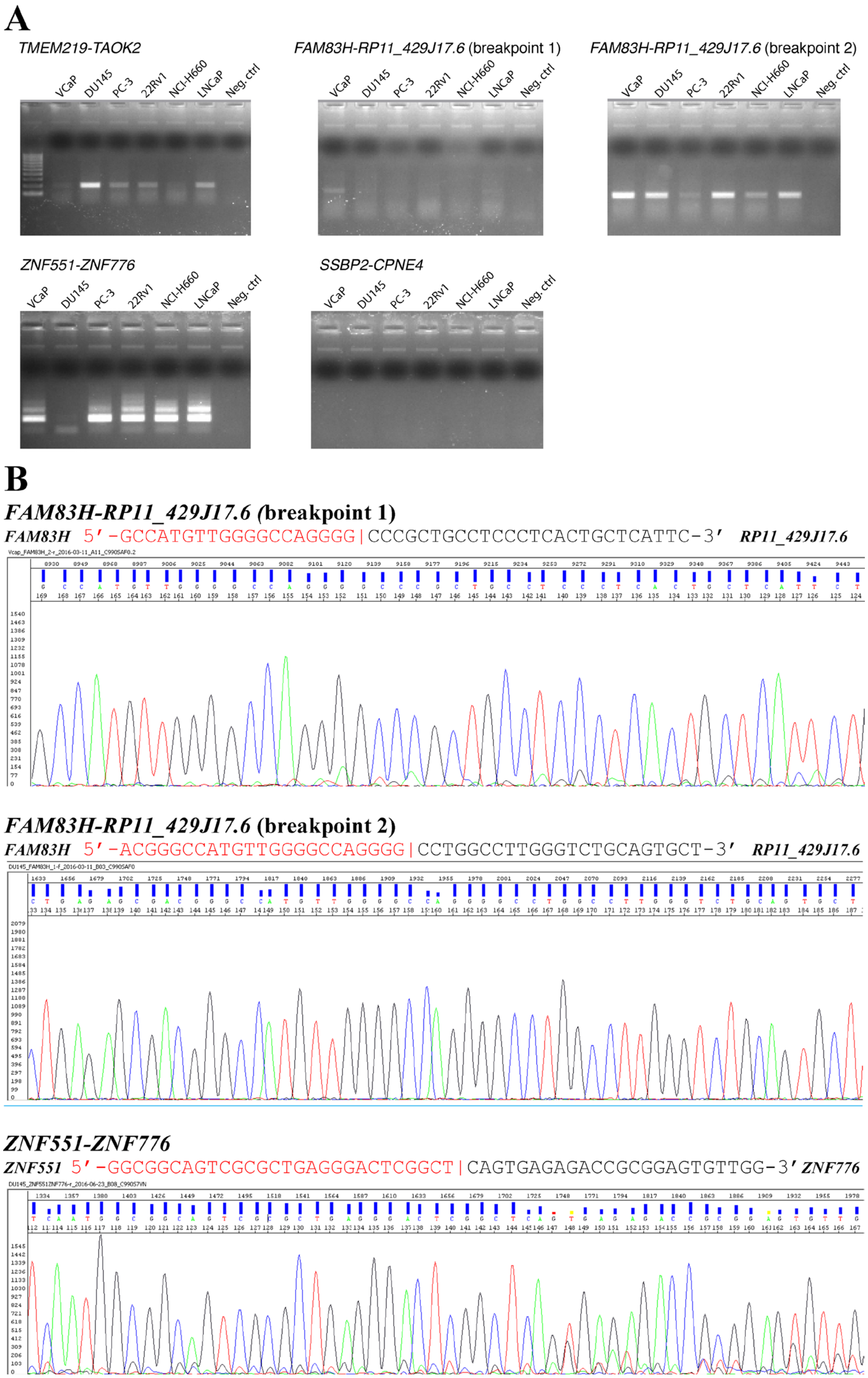

Supplementary Figure 9: RT-PCR validation of fusion transcripts. (**A**) RT-PCR products of five different fusion transcripts (matching results from Supplementary Table 2) in six prostate cancer cell lines. (**B**) Sanger sequencing with verification of the breakpoints of three fusion transcripts. For the fusion transcripts, the validated breakpoint sequences were identical in all positive cell lines, and one representative for each of the fusion transcripts is shown. Please note that for the *FAM83H-RP11_429J17.6* fusion transcript (breakpoint1), the gel bands are hardly visible on the photograph. However, Sanger sequencing of all of these produced the expected breakpoint sequences.

Supplementary Table 1: Novel fusion transcript in prostate cancer. See Supplementary_Table_1

Supplementary Table 2: RT-PCR and Sequencing results on six prostate cell lines

Fusion	Vcap	DU145	PC3	22Rv1	NCI-H660	LNCaP
TMEM219-TAOK2	v	v	v	v	x	v
FAM83H-RP11_429J17.6 (BP1)	v	v	v	v	x	v
FAM83H-RP11_429J17.6 (BP2)	v	v	v	v	v	v
ZNF551-ZNF776	v	v	v	v	v	v
SSBP2-CPNE4	x	x	x	x	x	x

BP1 and BP2 represent different breakpoints for fusion transcript FAM83H-RP11_429J17.6

v	the breakpoint was successfully validated by RT-PCR and Sanger sequencing (see Supplementary Figure 9)
x	no PCR product, or Sanger sequencing result does not match the breakpoint identified from RNA-seq data

Supplementary Table 3: Summary of functional characteristics of partner genes for novel fusion transcripts. See Supplementary_Table_3

Supplementary Table 4: Summary of read counts of RNA-seq data from TCGA and cancer cell line encyclopedia. See Supplementary_Table_4

Supplementary Table 5: Primers used for PCR of fusion candidates in prostate cancer cell lines

Primer name	Direction	Fusion	PCR	Primer sequence
ZNF551_F	Forward	*ZNF551-ZNF776*	Regular	5′- TCCACCTTCTGGGTTCAGTC-3′
ZNF776_R	Reverse	*ZNF551-ZNF776*	Regular	5′- GGACAGAGAAGTCGCGAAAG-3′
ZNF551_nestedF	Forward	*ZNF551-ZNF776*	Nested	5′- TTCTACACGACCCAACACTCC-3′
ZNF776_nestedR	Reverse	*ZNF551-ZNF776*	Nested	5′- GAAAGTGGGCCAGAGGTTCT-3′
FAMH83H_ALT2_F	Forward	*FAM83H-RP11_429J17.6 BP. 1*	Regular	5′- TGTAGTGAGGCGGCAGGTA-3′
FAM83HAS1_ALT2_R	Reverse	*FAM83H-RP11_429J17.6 BP. 1*	Regular	5′- CAGGGTCAGACCTGATGTGG-3′
FAM83H_F	Forward	*FAM83H-RP11_429J17.6 BP. 2*	Regular	5′- TCTTCAGGGCACAGGAAGTC-3
FAM83HAS1_2R	Reverse	*FAM83H-RP11_429J17.6 BP. 2*	Regular	5′- GAAGCACTGCAGACCCAAG-3′
SSBP2_2F	Forward	*SSBP2-CPNE4*	Regular	5′- TCAGAATCTTGCTCTGTCACC-3′
CPNE4_2R	Reverse	*SSBP2-CPNE4*	Regular	5′- ATTTCCAAGCTTATGGAGAAAAA-3′
SSBP2_F	Forward	*SSBP2-CPNE4*	Nested 1	5′- AGTGCAATGGTGTGATCTCG-3′
CPNE4_R	Reverse	*SSBP2-CPNE4*	Nested 1	5′- GCCTTCCTCTGGTAACTTTGG -3′
SSBP2_nestF	Forward	*SSBP2-CPNE4*	Nested 2	5′- GCTCACTGCAACCTTCACCT -3′
CPNE4_nestR	Reverse	*SSBP2-CPNE4*	Nested 2	5′- TGGAGACCCTCAGAGCTTTT-3′
TMEM219_F	Forward	*TMEM219-TAOK2*	Regular	5′- ACTGGCCTAGTTCCCGACTT -3′
TAOK2_R	Reverse	*TMEM219-TAOK2*	Regular	5′- GGCAAAGTATACGGCTCCAA -3′
**Custom designed assay sequences for TaqMan real-time PCR**				
Probe	CGGGAGAAGCAGCTC			
5′ primer	ACACTGGCCTCCCTCTACCA			
3′ primer	GGGTGATAGCACTGTCCATAGCA			
